# Changes in Smoking Habits and Behaviors Following the Introduction and Spread of Heated Tobacco Products in Japan and Its Effect on FEV_1_ Decline: A Longitudinal Cohort Study

**DOI:** 10.2188/jea.JE20210075

**Published:** 2022-04-05

**Authors:** Sei Harada, Mizuki Sata, Minako Matsumoto, Miho Iida, Ayano Takeuchi, Suzuka Kato, Aya Hirata, Kazuyo Kuwabara, Takuma Shibuki, Yoshiki Ishibashi, Daisuke Sugiyama, Tomonori Okamura, Toru Takebayashi

**Affiliations:** Department of Preventive Medicine and Public Health, Keio University School of Medicine, Tokyo, Japan

**Keywords:** heated tobacco products, electronic nicotine delivery devices, prevention, smoking caused disease

## Abstract

**Background:**

Heated tobacco product (HTP) use in Japan has rapidly increased. Despite this rapid spread, little is known about the health effects of HTP use. We conducted a longitudinal cohort study to investigate the change in smoking habits following the spread of HTP use and its effect on forced expiratory volume in 1 second (FEV_1_) decline.

**Methods:**

Participants consisted of a resident population (*n* = 2,612; mean age, 67.7 years) with FEV_1_ measurement in 2012–2014 and 2018–2019, and a worksite population (*n* = 722; mean age 49.3 years) without FEV_1_ data. Participants were categorized as combustible cigarette-only smokers, HTP-only users, dual users, past smokers, and never smokers. The association between smoking group and the change in smoking consumption over a mean 5.6 years was examined. Differences in annual FEV_1_ change between smoking groups were examined in the resident population.

**Results:**

Prevalence of HTP-only and dual users in 2018–2019 was 0.8% and 0.6% in the resident population, and 5.0% and 1.9% in the worksite population, respectively. The overall number of tobacco products smoked/used increased in dual users compared to baseline, but not in others. Annual FEV_1_ decline in dual users tended to be greater than that in cigarette-only smokers (16; 95% confidence interval, −34 to 2 mL/year after full adjustment). Participants switching to HTP-only use 1.7 years before had a similar FEV_1_ decline as cigarette-only smokers.

**Conclusions:**

HTP use, including dual use, is prevalent even in a rural region of Japan. Dual users appear to smoke/use tobacco products more and have a greater FEV_1_ decline. Tobacco policy should consider dual use as high-risk.

## INTRODUCTION

Heated tobacco product (HTP) use is rapidly increasing in Japan. Sales of HTPs commenced in 2014, ahead of the world, and 7.2% of Japanese men and 1.4% of women were reported to be users in 2018, versus 22.0% of men and 7.5% of women who used combustible cigarettes.^1^ Most HTP users previously smoked combustible cigarettes.^2^ This drastic change in the smoking habits of Japanese following the spread of HTPs should be appropriately reflected in tobacco control policies, including dual using. Given the now gradual adoption of HTPs in other countries,^3^^,^^4^ information on the effect of HTP spread will be valuable worldwide.

While some populations use HTP and combustible cigarettes concurrently, the actual number of such dual users is unclear, as studies have produced conflicting data. JASTIS^5^^,^^6^ and ITC Japan^2^^,^^7^ have shown that the majority of HTP users were concurrent cigarette smokers. On the other hand, the Japanese National Health and Nutrition Survey has reported the opposite.^8^ Therefore, providing data from an independent cohort study using face-to-face interviews would be of great significance. Moreover, the smoking behavior of HTP users, including dual users, is little known. From the perspective of tobacco control policy, an understanding of trends in smoking behavior is essential, including both nicotine dependence and stages in the transtheoretical model of smoking cessation.^9^

A second important issue in HTPs is their effect on health. Although levels of some toxic substances in combustible cigarette mainstream smoke are reported to be decreased in HTP aerosol,^10^^,^^11^ few studies have evaluated the effect of these changes in HTP users, so the health effects of HTPs remain unclear. Furthermore, even if HTPs reduce some of the harmful effects of combustible cigarettes when used alone, the health effects of dual use should also be considered.

Nevertheless, evaluation of the health effects of newly introduced smoking products is challenging. Long-term evaluation is required to determine whether the increases in cancer, cardiovascular disease, stroke, and COPD seen with combustible cigarette smoking^12^ can be replicated with HTPs. However, an adverse effect of smoking that is consistently observed over a relatively short period is a decrease in forced expiratory volume in 1 second (FEV_1_).^13^ To our knowledge, studies of the effect of HTP use on FEV_1_ are scarce.

The Tsuruoka Metabolomics Cohort Study (TMCS), a population-based cohort study in Japan, has collected longitudinal data on smoking habits and FEV_1_ from baseline in 2012, before HTPs were sold, to follow-up in 2018, after HTPs became popular. Here, we used these data to investigate the change in smoking habits following the introduction and spread of HTPs, to evaluate the behavior of HTP users, including dual users, and to examine the effect of HTP use on FEV_1_ decline.

## METHODS

### Study participants

This study was based on the TMCS, a prospective cohort study conducted in Tsuruoka City, located in the rural area of Yamagata Prefecture, Japan. The baseline survey of TMCS was conducted from fiscal year (April–March) 2012 to 2015 and enrolled 11,002 participants aged 35–74 years. Details on the cohort study are available elsewhere.^14^^–^^17^ Briefly, two different populations exist in TMCS, a resident population and a worksite population. The resident population consists of participants attending a municipal health check-up, and the worksite population consists of those from an occupational health check-up. The follow-up survey from the fiscal year 2018 to 2021 is currently ongoing for both populations. In this study, individuals from both the resident and worksite cohorts who participated in the follow-up survey at Shonai Health Management Center from April 2018 to June 2019 including detailed questionnaire for HTP use were selected for analysis (eFigure 1). To be precise, for resident population, a total of 4,760 participants were enrolled at the baseline surveys conducted in the fiscal years 2012 and 2014 at Shonai Health Management Center, and of these, 2,612 individuals who participated in the follow-up survey between April 2018 and March 2019 at the same facility were included in the present analysis. For the worksite population, 767 employees from three different workplaces in Tsuruoka City participated in the baseline survey between January 2013 and July 2014, of which 676 people participated in the follow-up survey between January and June 2019 and were subject to the present analysis. As an add-on to the worksite population, we recruited an additional 46 individuals under the age of 40 from the same worksites between March and June of 2019 to investigate the prevalence of smoking habits in younger generations after the market introduction of HTP. In total, the number of participants in the present study was 2,612 for the resident population and 722 for the worksite population (including the 46 additional younger participants). The demographics at baseline were comparable between those who participated in the 2018–2019 survey and those who did not (eTable 1).

The study was approved by the Medical Ethics Committee of the Keio University School of Medicine, Tokyo, Japan (Approval Nos. 20110264 and 20180336). All individual participants in this study provided written informed consent.

### Data collection

All data were obtained in the TMCS baseline survey from April 2012 to March 2015 and the follow-up survey from April 2018 to June 2019. Information on smoking habits, education, medical history and medication was collected through a standardized self-administered questionnaire and face-to-face interview by trained interviewers in both the baseline and follow-up surveys. Information on HTPs and e-cigarette smoking, nicotine dependence, and stage of behavioral change in smoking cessation was also collected at the follow-up survey.

Numbers of combustible cigarettes smoked and HTPs used daily were collected by product type (IQOS, Philip Morris Inter National Inc., USA; glo, British American Tobacco Plc., UK; and Ploom TECH, Japan Tobacco Inc., Japan). Photographs of each HTP type were shown to each participant by an interviewer to confirm one’s use of a particular product. The tobacco-containing insert of IQOS and glo is a stick, while Ploom TECH is a capsule.^2^ The use of one stick of IQOS or glo was considered equivalent to smoking one combustible cigarette. As for Ploom TECH, a single use was defined as using the device for 10 minutes or less, since smoking one cigarette typically lasts less than 10 minutes. Respondents were asked how many times a day they used their device based on this interpretation, and we assessed that single use of Ploom TECH device was equivalent to smoking one cigarette. Smoking history was also collected; namely, age at starting and quitting smoking. Smoking habits were categorized into the following five groups according to information from the follow-up survey: combustible cigarette-only smokers, HTP-only users, dual users, past smokers, and never smokers. No participant used electric nicotine delivery systems other than HTPs, such as e-cigarettes. Combustible cigarette-only smokers were identified as smokers who smoked at least one combustible cigarette per day, but no HTPs; and HTP-only users as participants who used HTPs at least once per day, but no combustible cigarettes. Participants with daily use of both a cigarette and an HTP were classified as dual users. All smokers at follow-up had started smoking before baseline; accordingly, the HTP-only and dual users had all smoked combustible cigarettes exclusively at baseline.

Nicotine dependence was evaluated using the Fagerstrom test for nicotine dependence (FTND).^18^^,^^19^ We classified an FTND score of 7–10 as high dependence, 3–6 as normal dependence, and 0–2 as low dependence. Stage of behavioral change in quitting smoking was assessed using the following stages: (1) pre-contemplation stage without interest in cessation (a smoker who answered “I am not interested in cessation”); (2) pre-contemplation stage with interest in cessation (“I am interested in cessation, but am not considering quitting within 6 months”); (3) contemplation stage (“I am considering quitting smoking within 6 months but am not ready to do so within 1 month”); and (4) preparation stage (“I am ready to quit smoking within 1 month”).^9^^,^^20^

Respiratory function was repeatedly tested in the resident population at the baseline and follow-up surveys. Of the 2,612 participants in the resident population, 2,475 were tested at both baseline and follow-up. FEV_1_ was measured by a trained clinical laboratory technician using an electric spirometer (Aurospiro AS7; Minato Medical Science, Yokohama, Japan). FEV_1_ change per year was calculated by subtracting FEV_1_ at baseline from FEV_1_ at follow-up, then dividing it by the individual’s follow-up period (mean 5.6; standard deviation [SD], 0.7 years).

### Statistical analysis

A linear regression model was used to examine the differences among smoking groups in change of the total number of tobacco products smoked or used per day from baseline to follow-up. This model included a dummy variable for the smoking group (combustible cigarette only smokers (reference category), HTP only users, and dual users) as the independent variable, and the number of tobacco products smoked/used per day at follow-up minus the number of those at baseline was used as the outcome variable. This analysis was conducted using the two populations combined. We used two models: model 1 was crude, and model 2 was adjusted for sex, age, population (resident or worksite) and the number of combustible cigarettes smoked per day at baseline (equal to total smoking of tobacco products per day at baseline). Sensitivity analysis that excluded Ploom TECH users (*n* = 8) was also conducted. A linear regression model was then developed to examine the differences between smoking groups in annual FEV_1_ change in the resident population. This model included a dummy variable for the smoking group (combustible cigarette only smokers (reference category), HTP only users, dual users, past smokers and never smokers) as the independent variable, and FEV_1_ change per year was used as the outcome variable. Participants who quit daily smoking before the baseline survey were defined as past smokers in the analysis for FEV_1_ change. Participants who quit daily smoking after baseline were excluded from this particular analysis (*n* = 143). Three models were used for this analysis. Model 1 was crude; model 2 was adjusted for sex and age; and model 3 was adjusted for sex, age, height, FEV_1_ at baseline, and the number of combustible cigarettes smoked per day at baseline. For past and never smokers, model 3 was conducted without adjustment for the number of combustible cigarettes. We also conducted a sensitivity analysis as model 4 that included the number of tobacco products smoked/used per day at follow-up instead of the number of combustible cigarettes smoked per day at baseline.

All statistical analyses were performed using R.3.6.2 (R Foundation for Statistical Computing, Vienna, Austria).

## RESULTS

### HTP usage and change in smoking habits

Prevalence of HTP-only users and dual users in 2018 was 0.8% and 0.6% in the resident population (mean age 67.7; SD, 7.1 years), and 5.0% and 1.9% in the worksite population (mean age 49.3; SD, 7.3 years), respectively (Table 1, Table 2, eTable 2, and eTable 3). The prevalence of combustible cigarette-only smokers was 8.9% in the resident and 8.6% in the worksite population. HTP-only users had switched from combustible cigarettes to HTPs a mean 1.66 (SD, 0.86) years before the follow-up survey, whereas dual users had started combining HTPs a mean 1.21 (SD, 1.01) years prior to follow-up. In both populations, HTP-only and dual users were younger than the other groups. HTP use was more frequent in the younger population (Table 3), and HTP users were accordingly much more prevalent in the worksite than the resident population. Dual users accounted for 29.2% of HTP users aged 40–49 years and over 40% of those aged 50–79 years in males. Among women using HTPs, 28.6% in their 40s and 33.3% in their 50s were dual users. IQOS was the most popular HTP brand. None of the participants were daily users of electronic nicotine delivery systems other than HTPs in either population.

**Table 1.** Characteristics of the resident population in the follow-up survey

		Overall	Combustible cigarette-only smoker	HTP-only user	Dual user	Past smoker^a^	Never smoker
*N* (%)		2,612 (100)	233 (8.9)	20 (0.8)	16 (0.6)	808 (30.9)	1,535 (58.8)
Sex, *n* (%)	male	1,177 (45.1)	205 (88.0)	18 (90.0)	15 (93.8)	715 (88.5)	224 (14.6)
	female	1,435 (54.9)	28 (12.0)	2 (10.0)	1 (6.2)	93 (11.5)	1,311 (85.4)
Age, years, mean (SD)		67.7 (7.1)	65.1 (7.1)	57.7 (9.2)	58.1 (8.5)	67.6 (7.5)	68.3 (6.7)
Total tobacco products number per day, mean (SD)		16.9 (6.8)	16.6 (6.6)	15.7 (6.1)	23.4 (8.3)	—	—
Cigarette number per day, mean (SD)		16.6 (6.7)	16.6 (6.6)	—	16.1 (8.4)	—	—
IQOS number per day, mean (SD)		10.1 (8.0)	—	13.9 (7.7)	5.3 (5.5)	—	—
glo number per day, mean (SD)		1.0 (3.6)	—	1.0 (4.5)	0.9 (2.1)	—	—
Ploom number per day, mean (SD)		0.9 (3.5)	—	0.8 (3.3)	1.1 (3.8)	—	—
Cigarette number per day at baseline, mean (SD)		18.4 (7.2)	18.2 (7.1)	18.3 (8.0)	21.8 (9.1)	—	—
Years of smoking, mean (SD)		30.0 (14.8)	43.8 (8.1)	36.3 (9.1)	38.1 (8.7)	25.7 (14.0)	—
Years of using HTPs, mean (SD)		1.5 (0.9)	—	1.7 (0.9)	1.1 (0.8)	—	—
FTND score, *n* (%)							
High (7–10)		21 (12.1)	16 (11.0)	3 (20.0)	2 (16.7)	—	—
Normal (3–6)		115 (66.5)	97 (66.9)	10 (66.7)	8 (66.7)	—	—
Low (0–2)		37 (21.4)	32 (22.1)	2 (13.3)	2 (16.7)	—	—
Stage of behavioral change in quitting smoking, *n* (%)							
pre-contemplation stage without interest		43 (24.3)	37 (24.7)	4 (26.7)	2 (16.7)	—	—
pre-contemplation stage with interest		93 (46.5)	77 (51.3)	8 (53.3)	8 (66.7)	—	—
contemplation stage		31 (15.5)	28 (18.7)	2 (13.3)	1 (8.3)	—	—
preparation stage		10 (5.6)	8 (5.3)	1 (6.7)	1 (8.3)	—	—
Educated, *n* (%)							
≤9 years		448 (17.3)	28 (12.1)	3 (15.8)	2 (12.5)	126 (15.8)	289 (19.0)
10–12 years		1,567 (60.6)	149 (64.2)	7 (36.8)	10 (62.5)	481 (60.1)	920 (60.5)
13–15 years		419 (16.2)	36 (15.5)	8 (42.1)	4 (25.0)	122 (15.2)	249 (16.4)
≥16 years		153 (5.9)	19 (8.2)	1 (5.3)	0 (0.0)	71 (8.9)	62 (4.1)
Height, cm, mean (SD)		158.4 (8.8)	165.3 (7.1)	169.2 (8.8)	165.7 (7.4)	164.2 (7.0)	154.1 (7.2)
FEV_1_, L, mean (SD)		2.20 (0.55)	2.42 (0.58)	2.90 (0.67)	2.69 (0.62)	2.49 (0.55)	2.00 (0.45)
FEV_1_ at baseline, L, mean (SD)		2.38 (0.58)	2.65 (0.60)	3.14 (0.60)	3.04 (0.62)	2.68 (0.57)	2.17 (0.47)
Follow-up period, years, mean (SD)		5.6 (0.7)	5.5 (0.9)	5.7 (0.9)	5.6 (0.8)	5.6 (0.8)	5.6 (0.8)

FEV_1_, forced expiratory volume in 1 second; FTND, Fagerstrom test for nicotine dependence; HTP, heated tobacco product; SD, standard deviation.

^a^Of the 808 past smokers, all had smoked cigarettes, and three had smoked/used both cigarettes and HTPs.

**Table 2.** Characteristics of the worksite population in the follow-up survey

		Overall	Combustible cigarette-only smoker	HTP-only user	Dual user	Past smoker^a^	Never smoker
*N* (%)		722 (100)	62 (8.6)	36 (5.0)	14 (1.9)	168 (23.3)	442 (61.2)
Sex, *n* (%)	male	250 (34.6)	38 (61.3)	28 (77.8)	11 (78.6)	104 (61.9)	69 (15.6)
	female	472 (65.4)	24 (38.7)	8 (22.2)	3 (21.4)	64 (38.1)	373 (84.4)
Age, years, mean (SD)		49.3 (7.3)	49.6 (7.4)	44.8 (8.3)	46.1 (9.0)	50.0 (7.2)	49.5 (7.0)
Total tobacco products number per day, mean (SD)		12.6 (7.0)	12.3 (8.0)	12.2 (4.4)	14.8 (8.0)	—	—
Cigarette number per day, mean (SD)		11.6 (7.6)	12.3 (8.0)	—	8.6 (4.4)	—	—
IQOS number per day, mean (SD)		8.2 (6.6)	—	10.0 (6.0)	3.6 (6.0)	—	—
glo number per day, mean (SD)		2.0 (5.0)	—	2.2 (5.3)	1.5 (4.1)	—	—
Ploom number per day, mean (SD)		0.3 (1.1)	—	0 (0.0)	1.1 (1.9)	—	—
Cigarette number per day at baseline, mean (SD)		14.4 (6.5)	13.4 (6.6)	14.5 (5.2)	14.0 (6.4)	—	—
Years of smoking, mean (SD)		19.2 (11.1)	27.8 (9.1)	22.9 (8.8)	26.1 (9.8)	14.6 (9.8)	—
Years of using HTPs, mean (SD)		1.52 (0.98)		1.6 (0.9)	1.3 (1.2)	—	—
FTND score, *n* (%)			—	—	—	—	—
High (7–10)		8 (7.3)	3 (5.0)	2 (5.6)	3 (21.4)	—	—
Normal (3–6)		49 (44.5)	22 (36.7)	20 (55.6)	7 (50.0)	—	—
Low (0–2)		53 (48.2)	35 (58.3)	14 (38.9)	4 (28.6)	—	—
Stage of behavioral change in quitting smoking, *n* (%)							
pre-contemplation stage without interest		21 (19.1)	9 (15.0)	9 (25.0)	3 (21.4)	—	—
pre-contemplation stage with interest		67 (60.9)	40 (66.7)	18 (50.0)	9 (64.3)	—	—
contemplation stage		15 (13.6)	6 (10.0)	7 (19.4)	2 (14.3)	—	—
preparation stage		7 (6.4)	5 (8.3)	2 (5.6)	0 (0.0)	—	—
Educated, *n* (%)							
≤9 years		1 (0.1)	0 (0.0)	0 (0.0)	0 (0.0)	0 (0.0)	1 (0.2)
10–12 years		214 (29.9)	21 (33.9)	16 (44.4)	5 (35.7)	48 (28.7)	124 (28.4)
13–15 years		349 (48.7)	23 (37.1)	13 (36.1)	7 (50.0)	66 (39.5)	240 (54.9)
≥16 years		152 (21.2)	18 (29.0)	7 (19.4)	2 (14.3)	53 (31.7)	72 (16.5)

FTND, Fagerstrom test for nicotine dependence; HTP, heated tobacco product; SD, standard deviation.

^a^Of the 168 past smokers, all had smoked cigarettes, and none had used HTPs.

**Table 3.** HTP use stratified by sex and age

	Combustible cigarette-only smoker	HTP-only user	Dual user	Past smoker	Never smoker	Dual/HTP users^a^
	
*N* (%)						
	
Overall						
34–39 years	2 (4.3)	10 (21.7)	3 (6.5)	5 (10.9)	26 (56.5)	23.1%
40–49 years	42 (10.2)	22 (5.4)	9 (2.2)	101 (24.6)	237 (57.7)	29.0%
50–59 years	54 (10.2)	13 (2.5)	10 (1.9)	161 (30.5)	290 (54.9)	43.5%
60–69 years	128 (10.6)	10 (0.8)	7 (0.6)	335 (27.6)	732 (60.4)	41.2%
70–79 years	69 (6.1)	1 (0.1)	1 (0.1)	371 (32.9)	685 (60.8)	50.0%
80 years	0 (0)	0 (0)	0 (0)	3 (30.0)	7 (70.0)	—
	
Male						
34–39 years	0 (0.0)	7 (25.9)	2 (7.4)	4 (14.8)	14 (51.9)	22.2%
40–49 years	27 (18.8)	17 (11.8)	7 (4.9)	55 (38.2)	38 (26.4)	29.2%
50–59 years	38 (18.1)	11 (5.2)	9 (4.3)	109 (51.9)	43 (20.5)	45.0%
60–69 years	112 (22.7)	10 (2.0)	7 (1.4)	290 (58.8)	74 (15.0)	41.2%
70–79 years	66 (12.0)	1 (0.2)	1 (0.2)	358 (65.3)	122 (22.3)	50.0%
80 years	0 (0.0)	0 (0.0)	0 (0.0)	3 (60.0)	2 (40.0)	—
	
Female						
34–39 years	2 (10.5)	3 (15.8)	1 (5.3)	1 (5.3)	12 (63.2)	25.0%
40–49 years	15 (5.6)	5 (1.9)	2 (0.7)	46 (17.2)	199 (74.5)	28.6%
50–59 years	16 (5.0)	2 (0.6)	1 (0.3)	52 (16.4)	247 (77.7)	33.3%
60–69 years	16 (2.2)	0 (0.0)	0 (0.0)	45 (6.3)	658 (91.5)	—
70–79 years	3 (0.5)	0 (0.0)	0 (0.0)	13 (2.2)	563 (97.2)	—
80 years	0 (0.0)	0 (0.0)	0 (0.0)	0 (0.0)	5 (100.0)	—

HTP, heated tobacco product.

^a^Proportion of dual users among all HTP users.

For combustible cigarette-only smokers and HTP-only users, the number of combustible cigarettes smoked per day at baseline and the number of tobacco products used per day at follow-up were comparable with a decreasing trend. Using combustible cigarette-only smokers as the reference group, −0.96 (95% confidence interval [CI], −2.58 to 0.67) products/day change was observed in HTP-only users after adjustment (Table 4). In contrast, in dual users, the total number of tobacco products (combustible cigarettes and HTPs) smoked and used per day increased, with a 3.12 (95% CI, 1.18–5.07) products/day change compared with combustible cigarette-only smokers after adjustment. Results were consistent after the exclusion of Ploom TECH users (−0.93; 95% CI, −2.57 to 0.70 products/day change in HTP only users and 3.18; 95% CI, 0.99–5.37 products/day in dual users). Although the number of combustible cigarettes smoked also decreased in dual users, the added number of HTPs led to an increase in the use of total number of smoking tobacco products.

**Table 4.** Differences between smoking groups in change of the total number of tobacco products smoked/used per day from baseline to follow-up (overall population)

	Differences in change of the total number of tobacco products smoked/used per day [95% CI]	

	Crude	Adjusted^a^
Combustible cigarette-only smoker	Ref	Ref
HTP only user	−0.79 [−2.39 to 0.81]	−0.96 [−2.58 to 0.67]
Dual user	2.71 [0.66–4.76]	3.12 [1.18–5.07]

CI, confidence interval; HTP, heated tobacco product.

^a^Adjusted for sex, age, number of cigarettes per day at baseline, and population.

The differences from cigarette-only smokers in change of the total number of tobacco products smoked/used per day from baseline to follow-up were estimated in the overall population.

### Nicotine dependence and stage of behavioral change in quitting smoking

For nicotine dependence, no difference was found between smoking groups in the resident population (χ^2^ test; *P* = 0.79). FTND score seemed to be marginally different in the worksite population (χ^2^ test; *P* = 0.05). Regarding the stage of behavioral change in quitting smoking, there were no obvious differences between smoking groups.

### FEV_1_

A linear regression model was used to examine the differences in annual FEV_1_ decline between smoking group (Table 5). Compared with combustible cigarette smokers, dual users showed greater FEV_1_ decline per year (−18; 95% CI, −35 to −1 mL/year in model 2, and −16; 95% CI, −34 to 2 mL/year in model 3), whereas HTP-only users had a similar reduction (1; 95% CI, −14 to 16 mL/year in model 2 and 6; 95% CI, −11 to 23 mL/year in model 3). Smaller FEV_1_ decline was observed for never and past smokers as compared to cigarette smokers. A sensitivity analysis was conducted by including the number of tobacco products smoked/used per day at follow-up as an adjusting covariate (model 4). Dual users still demonstrated a greater annual FEV_1_ decline than cigarette-only smokers, but their difference in the magnitude of the decline was smaller than observed in model 3.

**Table 5.** Differences between smoking groups in annual FEV_1_ change (mL/year) from baseline to follow-up (resident population only)

	Differences in annual FEV_1_ change—mL/year [95% CI]				Annual FEV_1_ change mL/year (SD)

	model 1 (crude)	model 2^a^	model 3	model 4	
Cigarette-only smoker	Ref	Ref	Ref	Ref	−44 (45)
HTP-only user	2 [−14 to 17]	1 [−14 to 16]	6 [−11 to 23]^b^	4 [−15 to 24]^d^	−43 (32)
Dual user	−18 [−36 to −1]	−18 [−35 to −1]	−16 [−34 to 2]^b^	−11 [−33 to 11]^d^	−63 (32)
Past smoker	10 [5–15]	10 [5–15]	14 [9–19]^c^	—	−34 (35)
Never smoker	13 [8–18]	3 [−2 to 9]	8 [3–14]^c^	—	−31 (32)

CI, confidence interval; FEV_1_, forced expiratory volume in 1 second; HTP, heated tobacco product; SD, standard deviation.

^a^Adjusted for sex and age.

^b^Adjusted for sex, age, height, FEV_1_ at baseline and number of cigarettes per day at baseline.

^c^Adjusted for sex, age, height and FEV_1_ at baseline.

^d^Adjusted for sex, age, height, FEV_1_ at baseline and number of tobacco products per day at follow-up.

The differences from cigarette-only smokers in FEV_1_ change per year (mL/year) were estimated in the resident population.

## DISCUSSION

This study showed that HTP use was prevalent even in a rural area of Japan. Among our participants, a prevalence of HTP use for men aged in their 40s, 50s, 60s, and 70s were 16.7%, 9.5%, 3.4%, and 0.4%, respectively, whereas for women, prevalence was 2.6% in their 40s, 0.9% in the 50s, and 0% in the 60s and 70s. These prevalence were similar to other surveys, which likely were heavily weighted towards urban areas.^1^^,^^8^ A population-based study conducted in Japan in 2018^1^ reported a prevalence of HTP use within the preceding 30 days of 13.3% of men and 2.5% of women aged 40–49 years, 7.7% and 2.3% in those aged 50–59 years, 5.6% and 0.2% in those aged 60–69, and 0.7% and 0% in those aged 70–79 years, respectively.^1^ The prevalence of HTP users in our study was largely consistent with this previous study, albeit higher in males and lower in females. This indicates that HTP use is prevalent in Japan, not only in urban areas but also in rural area, suggesting the need for nationwide measures for the spread of HTPs.

In this study, the proportion of dual use was 20–30% in HTP users aged 34–49 years, while it was nearly half among those aged 50 and above. An internet survey conducted in Japan in 2017 reported that the prevalence of HTP or e-cigarette use within the preceding 30 days was 4.7%. The dual use of combustible cigarettes and HTPs/e-cigarettes was 3.4%.^21^ The prevalence of dual users without e-cigarettes was not clear, but the proportion of dual users in HTP users was higher than in our study, at over 75% among participants aged 30 years and older. In that report, the prevalence of e-cigarette use appeared high, at 1.9%, despite the prohibition on the sale of e-cigarettes with nicotine by governmental regulation in Japan, indicating that participants likely misclassified these two new tobacco products, as noted by the authors. Such misclassification of HTP use is a major concern and has not been sufficiently addressed in the previous studies.^22^^,^^23^

Other surveys in Japan also showed a high proportion of dual users among HTP users,^2^^,^^5^^–^^7^ while that of the Japan National Health and Nutrition Survey in 2018 was similar to or slightly lower than our study.^8^ This conflict may be due to differences in sampling methods and questioning methods. In the Japan National Health and Nutrition Survey, random selection was conducted nationwide, but the questionnaire was limited in that the section on HTP use was not separated. Other nationwide surveys focused on HTP use, had detailed questionnaires on HTP use, but were conducted mainly through internet surveys.

The strength of this study is that misclassification was reduced as much as possible. We asked product-specific questions about HTP use, separate from the questionnaire for cigarette use, and confirmed each response through careful face-to-face interviews using photographs of the devices. Since the study was conducted in one rural region of Japan, the results are primarily generalizable for elderly residents and middle-aged workers in rural regions of Japan.

The number of smoking tobacco products used per day decreased over the 6 years of the observation period for both combustible cigarette smokers and HTP users, and the degree of reduction was comparable. This finding suggests that the shift to HTPs did not affect the number of tobacco products smoked. However, some combustible cigarette smokers started to use both combustible cigarettes and HTPs. These dual users tended to smoke a greater number of products than at baseline and showed a greater decline in FEV_1_ over the observation period compared to either the combustible cigarette only or HTP-only users. Considering many dual users among HTP users, the adverse health effects of HTP use and those of dual use should be considered when HTPs control policies are established.

It remains unclear whether switching from combustible cigarettes to HTPs causes a decrease in smoking consumption. In our study, smokers who switched to HTPs used a closely similar number of tobacco products to smokers who continued to smoke combustible cigarettes from baseline to follow-up. This result suggests that switching to HTPs does not contribute to a reduction in the number of products smoked, and therefore, the present study provides new insight into changing the habit of smoking. Further, our results also suggest that dual users tend to smoke more than they did prior to initiating the use of HTPs. One likely explanation for this is that HTP use might lead to a decrease in hesitation to smoke in public spaces, given that tobacco companies tend to emphasize the lower possibility of secondhand smoke exposure with HTP use. Indeed, 16 of our 30 dual users reported that they initially began to use HTPs because they would not bother the people around them (data not shown). It is possible that dual users smoked either combustible cigarettes or HTPs depending on the situation, resulting in more opportunities for the use of tobacco products than before. A second possibility is that dual users smoked more to compensate for the lower nicotine concentration delivered by HTPs. It is well known that lower nicotine cigarettes can produce compensatory smoking.^24^^–^^26^ Given that nicotine concentration in IQOS aerosol is reportedly only 84%^10^ or 71%^11^ of that in mainstream smoke of combustible cigarettes, compensatory smoking behavior might have occurred. A randomized controlled trial conducted by Philip Morris International of combustible cigarette smokers versus HTP-dominant users who used an almost equal number of tobacco products reported that urinary levels of nicotine metabolites after the 6-month trial were closely similar.^27^ If so, it can be considered that HTP users compensate for the reduced nicotine by using HTPs for longer or deeper than combustible cigarettes.

Nicotine dependence was closely consistent between combustible cigarette smokers and HTP-only users in this study. Switching to HTP use alone was not associated with nicotine dependence. It is possible that dual users had higher nicotine dependence than other smokers in the worksite population, but careful interpretation is needed because the sample size is small. Stages of behavioral change in quitting smoking did not differ among the three smoking groups. Only 5% of smokers were at the preparation stage, and most had little intention to quit, indicating that a shift to HTPs did not lead to smoking cessation. Further studies are needed to clarify effective interventions to quit HTP use.

We compared annual FEV_1_ change between combustible cigarette only, HTP only, and dual users to examine the health effect of daily HTP use during the few years after introduction to the market. Annual FEV_1_ change between combustible cigarette-only smokers and HTP-only users was comparable. This suggests that switch to HTP may not have affected respiratory function in the participants of this study. However, this should be interpreted carefully since the mean duration of HTP use was only 1.7 (SD, 0.9) years. In contrast, FEV_1_ declined substantially in dual users compared with combustible cigarette smokers or HTP-only users, in which the mean duration of dual using was 1.1 (SD, 0.8) years. Evidence for the health effects of smoking HTPs is markedly limited. Some toxic substances, such as tobacco-specific nitrosamines and polycyclic aromatic hydrocarbons, are reportedly reduced in IQOS aerosol, whereas other compounds, such as formaldehyde and nicotine, remain.^10^^,^^11^^,^^28^ Given the paucity of evaluation, it is not clear whether HTPs have fewer harmful effects than combustible cigarettes. A decline in FEV_1_ has been established as a short-term effect of the adverse effect of smoking cigarettes.^13^^,^^29^ This effect was also confirmed in our study. The annual decline in FEV_1_ among cigarette only smokers were 13 mL/year higher than never smokers, and comparable to previous studies. The results of a systematic review of 47 studies on the annual decline in FEV_1_ due to smoking showed that the annual decline in FEV_1_ was over 10 mL/year greater than in smokers compared with non-smokers.^13^ Although the sample size was small, our results showing that the decline in dual users was 18 mL/year greater than in cigarette-only smokers suggests that the adverse effects of smoking are more enhanced in elderly dual users. The differences in the magnitude of the FEV_1_ decline between cigarette-only smokers and dual users decreased by adjusting for the number of tobacco products smoked/used at follow-up. This result may suggest the more rapid decline in FEV_1_ observed in the dual users could be explained by the increase in smoking/using tobacco products after they started to use HTPs. Our present results were in contrast to those from a study conducted by Phillip Morris International, which reported that the use of HTPs improved lung function as measured by FEV_1_ compared to continuous use of combustible cigarettes for 6 months.^27^ Our study is significant in examining the association of dual use with respiratory function decline in a longitudinal cohort independent of the tobacco industry.

One of the limitations of this study is that it was conducted in a single rural area of Japan, primarily among elderly residents and middle-aged workers. The results should be generalized carefully. Another important limitation is that the analysis of respiratory function was conducted only among the resident population, so it included mainly elderly participants and a small number of HTP users. It should be noted that even when the number of HTP users was small and the duration of exposure was short, dual users showed a greater decline in respiratory function. Nevertheless, a longer follow-up study with a larger age group is needed for a more accurate discussion.

### Conclusions

HTP users are prevalent in Japan, particularly among younger generations and even in the rural area. In this study, around 30–50% of HTP users were dual users of both combustible cigarettes and HTPs among older adults and middle-aged workers in Japan. Dual users tended to smoke/use tobacco products more after they started to use HTPs. FEV_1_ appears to have declined more strongly in these dual users than in the combustible cigarette-only smokers and HTP-only users. Individuals who switched to HTP-only use a mean of 1.7 years before had a similar reduction in FEV_1_ as those who had exclusively smoked cigarettes. Although the adverse health effects of HTPs remain controversial, dual users should be marked as a high-risk group when tobacco policies for HTPs are considered.

## APPENDIX A. SUPPLEMENTARY DATA

eFigure 1. Flowchart of the survey. The detailed survey for heated tobacco product use was conducted 2018–2019. No participants had ever used heated tobacco products at the baseline survey in 2012–2015.

eTable 1. Comparison of the demographics at baseline of those who participated in the 2018–2019 survey and those who did not

eTable 2. Characteristics of the worksite population without add-on younger participants in the follow-up survey

eTable 3. Characteristics of the add-on younger participants for the worksite population in the follow-up survey
