# Supplementary material for: Changes in Smoking Habits and Behaviors Following the Introduction and Spread of Heated Tobacco Products in Japan and Its Effect on FEV1 Decline: A Longitudinal Cohort Study
Source: J Epidemiol. 2022 Apr 5;32(4):180–7. doi: 10.2188/jea.JE20210075 (PMC8918621; doi:10.2188/jea.JE20210075)
Supplement: Supplementary file 1 [file je-32-180-s001.pdf]

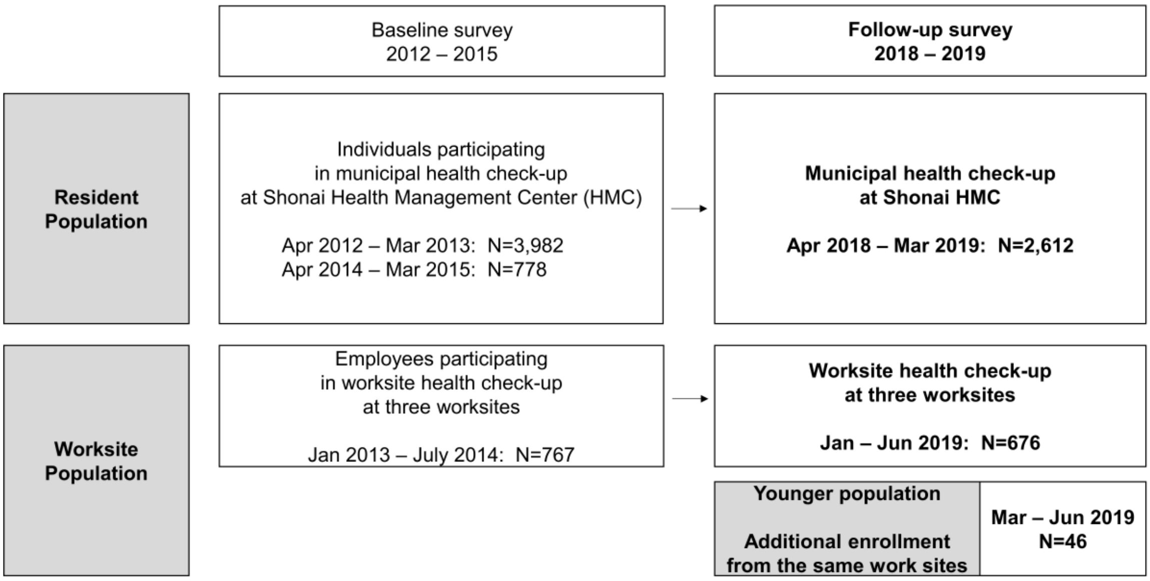

eFigure 1. Flowchart of the survey. The detailed survey for heated tobacco product use was conducted 2018–2019. No participants had ever used heated tobacco products at the baseline survey in 2012–2015.

eTable 1. Comparison of the demographics at baseline of those who participated in the 2018–2019 survey and those who did not

		Resident population		Worksite population	
2018–2019 survey		Participated	Not participated	Participated	Not Participated
N		2,612	2,148	676	91
Characteristics at baseline					
Sex (%)	male	45.1	46.9	32.8	35.5
	female	54.9	53.1	67.2	64.5
Age, years, mean (SD)		62.0 (7.1)	62.6 (8.1)	44.9 (6.6)	48.5 (9.5)
Smoking status, %					
Never smoker		58.8	55.7	61.8	54.7
Past smoker		27.1	28.0	21.1	24.3
Current smoker		14.2	16.3	17.6	21.1
Cigarette number per day, mean (SD)		18.4 (7.2)	19.8 (10.2)	14.2 (6.1)	14.1 (7.9)
Educated, %					
≤9 years		17.3	22.9	0.1	0.8
10–12 years		60.6	56.0	30.6	24.2
13–15 years		16.2	15.7	48.5	42.3
≥16 years		5.9	5.4	20.7	32.7
Height, cm, mean (SD)		158.4 (8.8)	158.9 (9.2)	163.2 (8.3)	163.0 (8.0)
FEV_1, L, mean (SD)		2.38 (0.58)	2.34 (0.62)	-	-

FEV_1, forced expiratory volume in 1 second; SD, standard deviation.

eTable 2. Characteristics of the worksite population without add-on younger participants in the follow-up survey

		Overall	Cigarette-only smoker	HTP-only user	Dual user	Past smoker	Never smoker
N (%)		676	60 (8.9)	26 (3.8)	9 (1.3)	163 (24.1)	418 (61.8)
Sex (%)	male	222 (32.8)	38 (63.3)	21 (80.8)	8 (88.9)	100 (61.3)	55 (13.2)
	female	454 (67.2)	22 (36.7)	5 (19.2)	1 (11.1)	63 (38.7)	363 (86.8)
Age, years, mean (SD)		50.3 (6.5)	50.1 (7.0)	48.5 (6.5)	50.6 (6.8)	50.4 (6.8)	50.4 (6.3)
Total tobacco products number per day, mean (SD)		12.9 (7.3)	12.4 (8.1)	12.3 (4.5)	18.2 (6.9)	-	-
Cigarette number per day		12.1 (7.7)	12.4 (8.1)	-	10.0 (3.5)	-	-
IQOS number per day		8.4 (6.7)	-	9.6 (6.2)	4.8 (7.1)	-	-
glo number per day		2.5 (5.7)	-	2.7 (5.9)	2.2 (5.1)	-	-
Ploom number per day		0.3 (1.0)	-	0 (0.0)	1.2 (1.8)	-	-
Cigarette number per day at baseline, mean (SD)		14.2 (6.1)	13.4 (6.6)	15.2 (5.0)	16.9 (4.6)	-	-
Years of smoking, mean (SD)		19.6 (11.3)	28.2 (8.9)	26.0 (8.4)	31.2 (6.6)	14.8 (9.9)	-
Years of using HTPs		1.7 (1.1)	-	1.7 (1.0)	1.6 (1.5)	-	-
FTND score, n (%)							
High (7–10)		7 (7.5)	3 (5.2)	1 (3.8)	3 (33.3)	-	-
Normal (3–6)		43 (46.2)	22 (37.9)	16 (61.5)	5 (55.6)	-	-
Low (0–2)		43 (46.2)	33 (56.9)	9 (34.6)	1 (11.1)	-	-
Stage of behavioral change in quitting smoking, n (%)							
pre-contemplation stage		18 (19.4)	9 (15.5)	7 (26.9)	2 (22.2)	-	-

without interest pre-contemplation stage	58 (62.4)	39 (67.2)	13 (50.0)	6 (66.7)	-	-
with interest contemplation stage	12 (12.9)	6 (10.3)	5 (19.2)	1 (11.1)	-	-
preparation stage	5 (5.4)	4 (6.9)	1 (3.8)	0 (0.0)	-	-
Educated, n (%)						
≤9 years	1 (0.1)	0 (0.0)	0 (0.0)	0 (0.0)	0 (0.0)	1 (0.2)
10–12 years	205 (30.6)	19 (31.7)	14 (53.8)	3 (33.3)	46 (28.4)	123 (29.8)
13–15 years	325 (48.5)	23 (38.3)	7 (26.9)	4 (44.4)	64 (39.5)	227 (55.0)
≥16 years	139 (20.7)	18 (30.0)	5 (19.2)	2 (22.2)	52 (32.1)	62 (15.0)
Follow-up period, years, mean (SD)	5.4 (0.5)	5.5 (0.5)	5.7 (0.4)	5.6 (0.5)	5.4 (0.5)	5.4 (0.5)

FTND, Fagerstrom test for nicotine dependence; HTP, heated tobacco product; SD, standard deviation.

eTable 3. Characteristics of the add-on younger participants for the worksite population in the follow-up survey

		Overall	Cigarette-only smoker	HTP-only user	Dual user	Past smoker	Never smoker
N (%)		46	2 (0.3)	10 (1.5)	5 (0.7)	5 (0.7)	24 (3.6)
Sex (%)	male	28 (60.9)	0 (0.0)	7 (70.0)	3 (60.0)	4 (80.0)	14 (58.3)
	female	18 (39.1)	2 (100.0)	3 (30.0)	2 (40.0)	1 (20.0)	10 (41.7)
Age, years, mean (SD)		35.7 (3.5)	35.0 (2.8)	35.2 (2.9)	38.0 (6.3)	36.0 (3.5)	35.4 (3.0)
Total tobacco products number per day, mean (SD)		10.5 (5.1)	7.5 (3.5)	12.0 (4.2)	8.8 (6.7)	-	-
Cigarette number per day		6.4 (4.5)	7.5 (3.5)	-	6.0 (5.1)	-	-
IQOS number per day		7.9 (6.6)	-	11.0 (5.7)	1.6 (2.1)	-	-
glo number per day		0.7 (2.6)	-	1.0 (3.2)	0.2 (0.5)	-	-
Ploom number per day		0.3 (1.3)	-	0.0 (0.0)	1.0 (2.2)	-	-
Years of smoking, mean (SD)		13.9 (5.5)	15.0 (2.8)	14.9 (3.3)	17.0 (7.9)	8.2 (3.6)	-
Years of using HTPs		1.2 (0.5)	-	1.4 (0.5)	0.8 (0.3)	-	-
FTND score, n (%)							
High (7–10)		1 (5.9)	0 (0.0)	1 (10.0)	0 (0.0)	-	-
Normal (3–6)		6 (35.3)	0 (0.0)	4 (40.0)	2 (40.0)	-	-
Low (0–2)		10 (58.8)	2 (100.0)	5 (50.0)	3 (60.0)	-	-
Stage of behavioral change in quitting smoking, n (%)							
pre-contemplation stage without interest		3 (17.6)	0 (0.0)	2 (20.0)	1 (20.0)	-	-
pre-contemplation stage		9 (52.9)	1 (50.0)	5 (50.0)	3 (60.0)	-	-

with interest						
contemplation stage	3 (17.6)	0 (0.0)	2 (20.0)	1 (20.0)	-	-
preparation stage	2 (11.8)	1 (50.0)	1 (10.0)	0 (0.0)	-	-
Educated, n (%)						
≤9 years	0 (0.0)	0 (0.0)	0 (0.0)	0 (0.0)	0 (0.0)	0 (0.0)
10–12 years	9 (19.6)	2 (100.0)	2 (20.0)	2 (40.0)	2 (40.0)	1 (4.2)
13–15 years	24 (52.2)	0 (0.0)	6 (60.0)	3 (60.0)	2 (40.0)	13 (54.2)
≥16 years	13 (28.3)	0 (0.0)	2 (20.0)	0 (0.0)	1 (20.0)	10 (41.7)

FTND, Fagerstrom test for nicotine dependence; HTP, heated tobacco product; SD, standard deviation.
